# Immediate side effects of Comirnaty COVID-19 vaccine: A nationwide survey of vaccinated people in Israel, December 2020 to March 2021

**DOI:** 10.2807/1560-7917.ES.2022.27.13.2100540

**Published:** 2022-03-31

**Authors:** Shirley Shapiro Ben David, Sharon Baruch Gez, Daniella Rahamim-Cohen, Na’ama Shamir-Stein, Uri Lerner, Anat Ekka Zohar

**Affiliations:** 1 Health Division, Maccabi Healthcare Services, Tel Aviv, Israel

**Keywords:** COVID-19, side effects, pregnancy, BNT162b2 vaccine, patient reporting, survey

## Abstract

**Background:**

COVID-19 vaccine safety is of major interest worldwide, since there is no prior experience with it. Israel was one of the first countries to widely use the Comirnaty vaccine.

**Aim:**

We aimed to assess the vaccine's short-term side effects directly from a large population and to predict influencing factors for self-reporting side effects.

**Methods:**

In a retrospective cohort study, we investigated self-reported systemic vaccine side-effects using electronic surveys sent to vaccinated individuals between 20 December 2020 and 11 March 2021, within 3 days following administration of the first and second dose. We determined predictors for reporting systemic side effects by logistic regression.

**Results:**

A total of 1,213,693 patients received at least one vaccine dose and 301,537 (24.8%) answered at least one survey. Among them, 68,162 (30.4%) and 89,854 (59.9%) individuals filled the first and the second dose surveys, respectively, and reported one or more side effects. Most common side effects were fatigue, headache and myalgia. Several respondents reported facial paraesthesia after first and second dose, respectively (n = 1,675; 0.7% and n = 1,601; 1.1%). Individuals younger than 40 years and women reported side effects more frequently than others, but pregnant women reported less. Pregnancy was a weak predictor for reporting any side effect in general and in particular fatigue, myalgia, headache, chills and fever.

**Conclusions:**

We found further support for minor short-term side effects, within 3 days of receiving the Comirnaty vaccine. These findings from vaccine recipients in general and pregnant women in particular can improve vaccine acceptance.

## Introduction

The coronavirus disease (COVID-19) pandemic has caused morbidity and mortality in Israel and a worldwide, large-scale health challenge on hospital and community settings [1]. On 20 December 2020, Israel initiated a large COVID-19 vaccination campaign, during which the Comirnaty (BNT162b2, BioNTech/Pfizer, Mainz, Germany/New York, United States (US)) vaccine was administered [2]. In Israel, the public health system comprises only four not-for-profit health maintenance organisations (HMO), and vaccinations were widely distributed by these HMO through special vaccination centres and local nurses' clinics. The whole Israeli population 16 years and older was eligible to receive the vaccine according to a prioritisation system determined by the Israeli Ministry of Health (MoH) [3]. Senior citizens 60 years and older and healthcare workers were the first populations to get vaccinated in Israel, starting from 20 December 2020. On 4 February 2021, vaccines were offered to all citizens 16 years and older.

The safety of the Comirnaty vaccine is of major interest worldwide since there is no prior experience with the vaccine. Post-marketing safety surveillance is critical for patient health and safety, for all medications and vaccines [4]. While most reports concerning side effects rely primarily on manufacturers and healthcare professionals, there may be a discrepancy between those reports and the reports from the vaccinated individuals [5]. Specifically, data concerning immediate systemic side effects, within a week, among pregnant women is scarce [6] because they were excluded from the initial COVID-19 vaccine trials [7]. Transparent information about potential vaccine-related side effects is of paramount importance to increase confidence in vaccines [8,9].

In this study, our first aim was to examine the safety of the Comirnaty vaccine with regard to short-term side effects within 3 days as directly reported from a large population shortly after receiving a first and/or second dose of the vaccine. Our second aim was to evaluate predictors for reporting side effects among vaccinated patients overall and among pregnant women in particular.

## Methods

This investigation was an observational cross-sectional study consisting of data collected through an electronic survey of vaccinated individuals.

### Questionnaire development

An online survey was developed as part of COVID-19 vaccine safety monitoring by Maccabi Healthcare Services (MHS). MHS is the second largest HMO in Israel, serving more than 2.5 million citizens, representing a quarter of the Israeli population. The questionnaire was composed of three questions regarding potential systemic symptoms after receiving a dose of the Comirnaty vaccine and the respondent's age and sex (see Supplement).

Each questionnaire included a predetermined list of symptoms, and participants were asked to choose whether they experienced any of them or not. The order of these symptoms changed randomly for every recipient of the survey. Upon completion of the questionnaire, respondents could add information on other symptoms in free text form.

The predetermined list of symptoms was based on common side effects published in the phase III trial of the Comirnaty vaccine [7]. During the first 2 weeks of the survey, all supplemental information in the free text form was manually reviewed on a daily basis in order to identify additional common systemic side effects that were not included in the original questionnaire. For some of the side effects reported (i.e. hoarseness, cough, eye irritation, facial swelling, rash), as defined by the MHS joint risk management committee, the respondents were instructed to contact their attending physician.

### Survey administration

A link to the electronic survey was distributed by text messages to adults 18 years and older who are eligible by national law to answer surveys, 3 days after receiving a dose of the Comirnaty vaccine, between 20 December 2020 and 11 March 2021. Inclusion criteria were age 18–85 years, a valid cell phone number or email in MHS systems and no prior refusal to participate in electronic surveys. Individuals over the age of 75 years or without access to text messages received the survey via electronic mail. The invitation to participate in the survey was sent only once and was available in Hebrew and Arabic, the official languages of Israel. No follow-up was preformed to determine whether the reported side effects were transient or long-lasting. The link to the survey was valid for only 48 h. The response to the questionnaire was voluntary and without remuneration. The questionnaires were available in five languages: Hebrew, Arabic, Russian, French and English.

### Data collection

The MHS has a nationwide centralised database spanning over 20 years. All surveys were linked to the Electronic Health Record by a unique patient identifier. Pregnancy status, diabetes, immunosuppression and oncological-related background were identified from MHS registries. For our analyses, we took data for age and sex from the MHS registries, although this information was also collected in the questionnaires. These registries are based on validated inclusion and exclusion criteria (considering coded diagnoses, treatments and laboratory findings, where applicable). The registries are continuously and retrospectively (since 1998) updated based on each patient’s central medical record. Residential socioeconomic status was based on a score ranked from 1 (lowest) to 10 derived by the Israel Central Bureau of Statistics [10]. Ethnical groups included Arabs, ultra-orthodox Jews and general (not ultra-orthodox) Jews.

### Data analysis

Only surveys that included data regarding short-term side effects were included in the analysis. Association between respondents' characteristics and reporting side effects was estimated firstly by univariate analysis. Data were weighted by age group and sex to make the sample representative of the general vaccinated population registered at MHS [11].

Chi-squared test was used for categorical variables, and t-test for continuous variables. Furthermore, we performed a multivariable logistic regression using a binary logistic regression model applied to the survey respondents. 'Enter' method was used for including variables in the model. Regression was presented as adjusted odds ratios (OR) and 95% confidence interval (CI).

### Pregnant respondents

In order to evaluate the association between pregnancy status and reporting specific symptoms, we set up a matched case–control study between pregnant and non-pregnant female respondents in 1:4 ratio, by age, socioeconomic status and ethnicity. The OR was calculated by the prevalence of systemic side effects. Adjusted OR was calculated by adjusting for comorbidities (immunosuppression, body mass index > 35, cancer and diabetes).

All statistical analyses were performed with the Statistical Package for the Social Sciences (SPSS) version 25 (IBM Corp., New York, US).

### Ethical considerations

The study was approved by the MHS institutional review board (IRB) (0029–21-MHS). Informed consent was waived by the IRB, as all identifying details of the participants were removed before the computational analysis.

## Results

**Figure 1:**
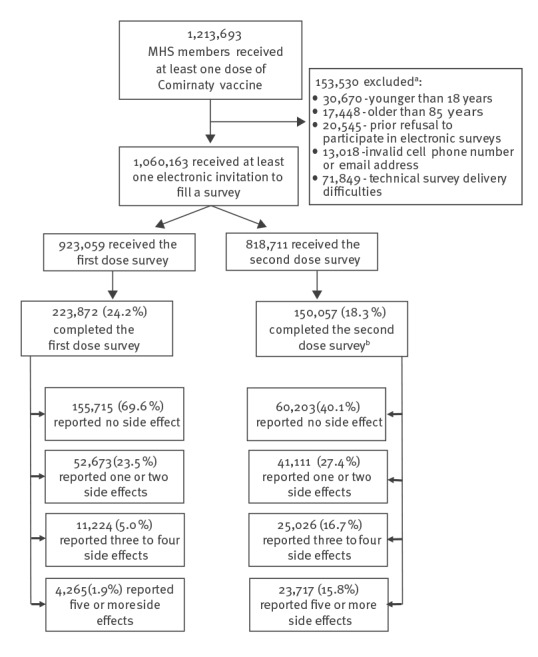
Flowchart of cohort creation, study of side effects after COVID-19 vaccination with Comirnaty, Israel, December 2020–March 2021 (n = 1,213,693)

COVID-19: coronavirus disease; MHS: Maccabi Healthcare Services.

^a^ Inclusion criteria were age 18–85 years, a valid cell phone number or email in the MHS systems and no prior refusal to participate in electronic surveys.

^b^ 72,392 individuals filled the first and the second survey.

By 11 March 2021, MHS had administered at least one Comirnaty vaccine dose to 1,213,693 of its members, representing 70.2% of the vaccine target population. Their mean age was 47.9 years (± standard deviation (SD): 17.5). Just over half of them (51.8%) were women (Table 1). Of 1,060,163 who met the inclusion criteria and received the electronic survey invitation (Figure 1), information on possible side effects of the vaccine was available for 301,537 (28.4%) individuals, who answered at least one-self-reported survey. The response rate for the first and second survey was 24.2% and 18.3%, respectively. A total of 106,291 and 106,622 individuals older than75 years or without access to text messages received the survey via electronic mail after, respectively, the first and second vaccine dose. Among them, respectively 32,242 (30.3%) and 11,241 (10.5%) filled the first and second survey. The survey respondents' mean age was 49.4 (± SD: 15.7) years.

**Table 1:** Characteristics of the general recipients of Comirnaty vaccine against COVID-19 and survey respondents, Israel, December 2020–March 2021 (n = 1,213,693)

	All vaccine recipients n = 1,213,693		Survey respondents n = 301,537	
	n	%	n	%
Sex*				
Male	584,997	48.2	141,037	46.8
Female	628,696	51.8	160,500	53.2
Age group (years)*				
18–40	443,112	36.5	90,230	29.9
41–65	549,047	45.2	157,320	52.2
> 65	221,534	18.3	53,987	17.9
Comorbidities (one or more)^a^				
Immunosuppression*	28,775	2.4	7,971	2.6
Morbid obesity (BMI ≥ 35)*	70,172	5.8	16,774	5.6
Cancer*	97,909	8.1	30,199	10.0
Diabetes*	117,254	9.7	26,595	8.8
Pregnancy^b,^*	10,668	0.9	2,234	0.7
Ethnicity/sector*				
Arabs	54,060	4.5	5,101	1.7
Jewish ultra-orthodox	59,164	4.9	10,072	3.3
Jewish non-orthodox	1,100,469	90.7	286,364	95.0
Socioeconomic status^b,^*				
Low	182,006	15.0	24,967	8.3
Medium	602,927	49.7	141,206	46.8
High	428,649	35.3	135,364	44.9
Smoking*				
Non-smoker	997,958	82.2	258,291	85.7
Previous smoker	17,647	1.5	4,494	1.5
Smoker	156,583	12.9	30,285	10.0
Unknown	41,505	3.4	8,467	2.8

BMI: body mass index (kg/m^2^); COVID-19: coronavirus disease; MHS: Maccabi Healthcare Services.

^a^ Identified from MHS registries.

^b^ Residential socioeconomic status was based on a score ranked from 1 (lowest) to 10 derived by the Israel Central Bureau of Statistics [10].

* All background characteristics were statistically significant (p < 0.001).

Comparison between background characteristics of the general vaccine population and the survey respondents. Respondents who answered at least one of the two surveys (after the first or the second vaccine dose). Inclusion criteria were 18–85 years-old, a valid cell phone number or email in the MHS systems and no prior refusal to participate in electronic surveys. Data are presented as absolute (relative) frequency or mean (± SD). Chi-squared test was used for categorical variables.

Among the 301,537 respondents, 168,861 (56.0%), reported one or more side effects. Side effects were more frequently reported in the second dose survey (89,854/150,057; 59.9%) compared with the first dose survey (68,162/223,872; 30.4%). Most of the respondents who did not report any side effect in the first dose survey (n = 103,257/155,715; 66.3%) did not fill in the second dose survey, while most of the respondents who filled in only the second dose survey (n = 54,284/77,665; 69.9%) reported having a side effect.

**Figure 2:**
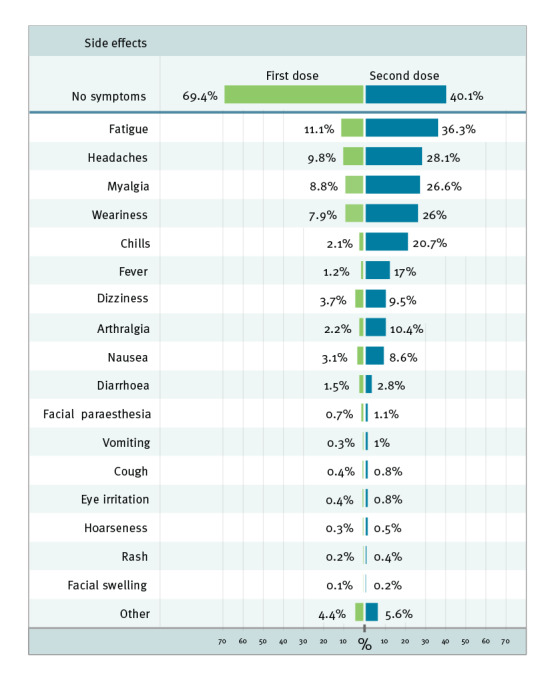
Reported side effects in the first (n = 223,872) and second dose (n = 150,057) surveys after COVID-19 vaccination with Comirnaty, Israel, December 2020–March 2021

The symptoms most frequently reported among all respondents for the respective doses were fatigue (n = 24,866; 11.1% and n = 54,417; 36.3% for first and second dose, respectively), headache (n = 21,974; 9.8% and n = 42,134; 28.1%) and myalgia (n = 19,791; 8.8% and n = 39,949; 26.6%) (Figure 2). Facial paraesthesia was reported by 1,675 (0.7%) and 1,601 (1.1%) of the first and second dose survey respondents, respectively. All reported side effects were more frequent after receipt of the second dose than after the first one, among all the respondents. Most of the respondents who reported a side effect (52,673/68,162; 77.3% for the first and 41,111/89,854; 45.8% for the second dose survey) reported only one or two symptoms. A third of the respondents to the second dose survey, 48,743 (32.5%), reported three symptoms or more, compared with 15,489 (6.9%) for the first dose (Figure 1). A total of 6,468 individuals used the free text response to add supplemental information beyond the symptoms listed in the questionnaires. About a third of them (n = 2,262) reported injection-site pain, and most expanded on one of the previously listed symptoms, giving descriptions such as 'a severe headache' or 'a productive cough'. No unexpected side effects were reported.

COVID-19: coronavirus disease.

Participants could indicate multiple symptoms. Proportions were weighted by age group and sex to make the sample representative of the general vaccinated population in the Maccabi Healthcare Services.

**Figure 3:**
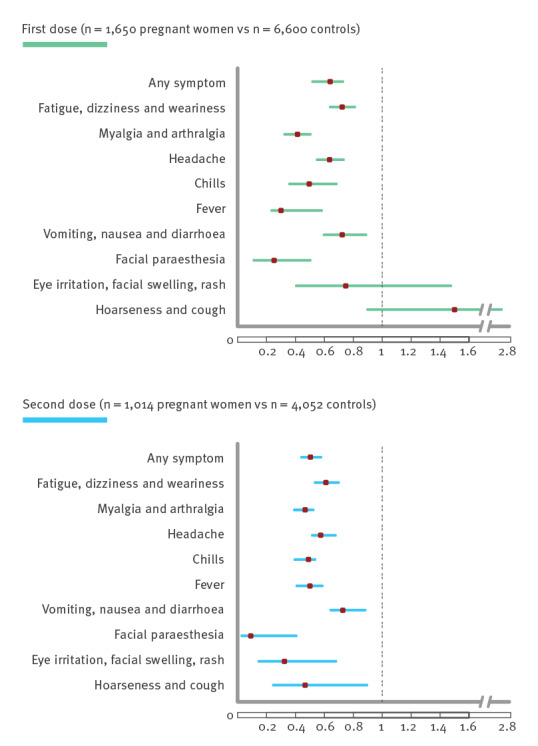
Adjusted odds ratios of reporting side effect comparing pregnant respondents with matched female controls, study on side effects after COVID-19 vaccination with Comirnaty, Israel, December 2020–March 2021

Crude-level correlations between reporting side effects and potential predictors found a strong link between age under 40 years, female sex and the tendency to report side effects in both the first and the second dose survey (Table 2), whereas comorbidities, particularly cancer and diabetes, were not correlated. Young age and female sex remained the strongest predictors in multivariate regression analysis (Table 3). Comorbidities (diabetes, morbid obesity, immunosuppression or cancer) were found as inconsistent predictors to report side effects in the first and second dose surveys. Comparison between pregnant women and their matched controls by age and ethnicity revealed that pregnant women reported symptoms less frequently than their controls (see the Supplement for details on this comparison, for all symptoms and each survey). Symptoms were also more commonly reported by pregnant women after the second dose than the first. Pregnancy was consistently less associated with reporting side effects than any other variables in univariate and multivariate analysis and in both surveys. Further comparison between pregnant female respondents and their matched controls found pregnancy to be a weak predictor for reporting any side effect in general and specifically fatigue, myalgia, headache, chills and fever (Figure 3). This pattern persisted over the two surveys.

**Table 2:** Comparison between respondents' characteristics and reporting side effects after COVID-19 vaccination with Comirnaty, Israel, December 2020–March 2021

	First dose				Second dose			
	One or more systemic side effects^a^ n = 66,596		No systemic side effects^a^ n = 157,276		One or more systemic side effects^a^ n = 88,899		No systemic side effects^a^ n = 61,158	
	n	%	n	%	n	%	n	%
Sex								
Male	23,628	35.5	81,324	51.7	54,080	60.8	25,723	42.1
Female	42,968	64.5	75,952	48.3	34,819	39.2	35,435	57.9
Age group (years)								
18–40	25,421	38.2	39,219	24.9	29,398	33.1	10,295	16.8
41–65	33,343	50.1	82,379	52.4	49,290	55.4	32,655	53.4
> 65	7,832	11.8	35,678	22.7	10,211	11.5	18,208	29.8
Ethnicity/sector								
Arabs	1,231	1.8	2,713	1.7	1,037	1.2	872	1.4
Jewish ultra-orthodox	2,764	4.2	5,014	3.2	2,805	3.2	1,853	3
Jewish non-orthodox	62,601	94	149,549	95.1	85,057	95.7	58,433	95.5
Socioeconomic status^b^								
Low	6,329	9.5	12,394	7.9	6,572	7.4	4,563	7.5
Medium	32,428	48.7	72,035	45.8	41,245	46.4	27,590	45.1
High	27,839	41.8	72,847	46.3	41,082	46.2	29,005	47.4
Pregnancy^a^	591	0.9	1,059	0.7	1,980	2.2	1,998	3.3
Comorbidities (one or more)^a^								
Immunosuppression	1,682	2.5	4,533	2.9	4,857	5.5	3,658	6
Morbid obesity (BMI ≥ 35)	4,050	6.1	8,542	5.4	7,608	8.6	8,522	13.9
Cancer	5,535	8.3	18,364	11.7	2,556	2.9	7,985	13.1
Diabetes	4,572	6.9	16,168	10.3	671	0.8	343	0.6
Smoking								
Non smoker	57,248	86	135,854	86.4	77,290	86.9	52,198	85.3
Previous smoker	945	1.4	2,407	1.5	1,173	1.3	1,019	1.7
Smoker	6,638	10	15,240	9.7	8,091	9.1	6,542	10.7
Unknown	1,765	2.7	3,775	2.4	2,345	2.6	1,399	2.3

BMI: body mass index (kg/m^2^); COVID-19: coronavirus disease.

^a^ Identified from Maccabi Healthcare Services registries.

^b^ Residential socioeconomic status was based on a score ranked from 1 (lowest) to 10 derived by the Israel Central Bureau of Statistics [10].

Chi-squared test was used for categorical variables, comparing side effects those after the first dose with no side effects. All factors reported were statistically significant (p < 0.001).

**Table 3:** Predictors for reporting side effects after COVID-19 vaccination with Comirnaty, by multivariate analysis, Israel, December 2020–March 2021

	First dose			Second dose		
	AOR	95% CI	p value	AOR	95% CI	p value
Age (years)^a^	0.976	0.976–0.977	< 0.001	0.963	0.962–0.964	< 0.001
Sex						
Male	Reference					
Female	1.92	1.89–1.96	< 0.001	2.13	2.08–2.17	< 0.001
Comorbidities						
No comorbidity	Reference					
Immunosuppression	1.03	0.97–1.09	0.312	0.86	0.80–0.92	< 0.001
BMI > 35	1.18	1.13–1.23	< 0.001	0.96	0.92–1.01	0.096
Cancer	0.97	0.93–0.99	0.039	0.95	0.92–0.98	0.004
Diabetes	1.01	0.97–1.05	0.610	0.82	0.79–0.85	< 0.001
Pregnancy status	0.65	0.59–0.72	< 0.001	0.44	0.39–0.51	< 0.001

AOR: adjusted odds ratio; BMI: body mass index (kg/m^2^); CI: confidence interval; COVID-19: coronavirus disease.

^a^ Age was a continuous variable, increase or decrease per year.

Multivariate logistic regression model. Comorbidities and pregnancy status were identified from Maccabi Healthcare Services registries.

COVID-19: coronavirus disease.

Calculated odds ratios are presented along with indicating 95% confidence intervals. Non-pregnant controls were female respondents matched by age and ethnicity. When compared with pregnant female respondents, the ratio was 4:1. Adjusted odds ratios were calculated by adjusting for comorbidities (immunosuppression, body mass index > 35, cancer and diabetes).

## Discussion

This large-scale study in a real-world community setting offers important insights into short-term systemic events within 3 days after receiving the Comirnaty vaccine. Data gathered directly from the vaccine recipients revealed overall not serious systemic side effects. After controlling for multiple factors, such as age, sex, comorbidities (diabetes, immunosuppression, cancer, obesity) and pregnancy status, adverse effects were more frequently reported in younger individuals and women, but less frequently among pregnant women (adjusted for age and ethnicity).

Passive reporting of vaccine recipients' side effects by healthcare professionals is the primary tool used for post-marketing surveillance (pharmacovigilance) of vaccine safety [5]. However, vaccine recipients play an essential part in subjectively identifying side effects and providing information about their incidence [12]. The rate of reports on systemic side effects from passive surveillance gathered by the Israeli MOH was substantially lower than in our study, recording the occurrence of fatigue and headache in, respectively, 0.3% and 0.25% of individuals after the second dose of vaccine [13], compared with 36.3% and 28.1% of the respondents in this survey. This discrepancy may indicate an under-reporting pattern of mild symptoms that do not require medical follow-up and do not interfere with daily routines. Our results confirm the expected adverse effects as reported in the pharmacovigilance reports but emphasise the value of the active response surveys as a research tool. The prevalence of systemic side effects of the Comirnaty vaccine was lower among the survey respondents than reported in the phase III trial, i.e. fatigue and headache after the second dose were reported by 59% and 52%, respectively, among younger vaccine recipients and by 51% and 39% among older recipients in the clinical trial [7]. This may reflect an overall high level of motivation to participate in the survey even without any symptoms to report. It may also result from more stringent reporting in the clinical trial compared with the suboptimal reporting in the real-world scenario. Nevertheless, our results are in concordance with another real-world study preformed in the United Kingdom [14].

Concerns about vaccine safety and potential side effects are part of the main barriers against vaccine acceptance [15-17]. In order to avoid over-heterogeneity of systemic adverse events, the survey was composed of built-in options of side effects and additional free text to maximise transparency of the side effect reports. We found that fatigue, headache and myalgia were the most commonly reported systemic events, and they were more prevalent among the second-dose and younger vaccine recipients. Notably, a unique side effect that had not been reported before [18], facial paraesthesia, was reported in 0.7% and 1.1% of respondents after the first and the second dose, respectively. This specific complaint was also reported to various media outlets by vaccinated individuals and was the topic of many online debates. It was also meticulously discussed by the appointed committee that oversees the vaccination campaign in Israel, and the incidence rate was found to be lower than that expected within the general population [13]. No other unexpected phenomenon was reported by the respondents using free text. These observations confirm that reports of mild immediate side effects, derived directly from the vaccinated individuals, can be used as additional relevant information when counselling patients about vaccination.

We found that women had twice the probability of reporting side effects compared with men and the rate of reporting increased inversely with age. This pattern may be behavioural or may reflect a more robust immune response [19,20]. Interestingly, there was no association between several comorbidities and reporting side effects. Considering that older age and certain underlying medical conditions are major risk factors for severe illness from COVID-19 [21,22], it is particularly important in this group of patients to underline that side effects are mild and therefore not an indication to avoid vaccination. Interestingly, we found that the fact of being pregnant at vaccination reduced the reporting ratio of all symptoms when compared with randomly matched controls. Different pharmacokinetics, masking of side effects by pregnancy symptoms or minimal expectation of side effects following vaccination [23] might be possible explanations. Skjefte et al. found that one of the top reasons for women to decline vaccination during pregnancy is their reluctance to expose their developing baby to any possible harmful side effects [24]. Comparison between pregnant women and their matched controls revealed fewer self-reported adverse reactions among pregnant women. Fever in particular, associated with adverse pregnancy outcome [25], was under-reported. Although pregnant women's opinion about the vaccination may be influenced mainly by severe and rare outcomes which might not be expressed in this survey, this information, confirming the immediate effects directly from the women, may contribute to increase vaccine confidence.

The strengths of our study are firstly its methodology, a survey systematically sent to most vaccinated individuals, which provides a valuable notification and reporting tool that can reach a large population. Other strengths include a large sample size and a 12-week study period, used to maximise the heterogeneity of people receiving at least one vaccine dose. The connection to the respondents' medical records, which gives validity to their background characteristics without relying only on their reports is also a strength.

Several limitations should be considered for this study. Firstly, the questionnaire did not provide information on fatal outcomes or outcomes that prevented the patient from filling out the questionnaire, and the number of respondents was not large enough to identify rare events. Also, with the time frame in which the survey was conducted, as questionnaires were sent and completed a few days after the vaccine uptake, long-term outcomes would not be observed by this study design and the duration of reported symptoms was not obtained. As with all public surveys, ours was a snapshot taken at a given point in time. Secondly, there may have been self-selection bias as participation was voluntarily and may have captured people more interested or willing to participate in online studies who may not be representative of the general population. Thirdly, the findings may not be representative of all vaccine recipients nationally as minorities and people of low socioeconomic status were less represented among the respondents. Finally, an electronic format may not be appropriate for some segments of the population such as elderly people [26] or those with major comorbidities because of physical or cognitive barriers; therefore, a non-electronic format or other options may improve representability of the survey.

## Conclusions

In this extensive voluntary survey regarding short-term side effects of the Comirnaty vaccine, we found further support for the short-term safety of the vaccine. Systemic side effects after vaccination were less common in a real-world community setting than reported in phase III trial, but notably higher than reported by the Israeli MoH. We found that women had twice the probability of reporting side effects compared with men and the rate of reporting increased inversely with age. Pregnant women reported side effect less frequently than their matched controls. These findings present valuable information for individuals considering vaccination and for healthcare professionals and can contribute to increasing vaccine confidence. Continued monitoring of long-term side effect merits further studies. 

## Supplementary Data

524000 Supplement
