# Supplementary material for: Immediate side effects of Comirnaty COVID-19 vaccine: A nationwide survey of vaccinated people in Israel, December 2020 to March 2021
Source: Euro Surveill. 2022 Mar 31;27(13):2100540. doi: 10.2807/1560-7917.ES.2022.27.13.2100540 (PMC8973016; doi:10.2807/1560-7917.ES.2022.27.13.2100540)
Supplement: Supplement [file 21-00540_SHAPIRO_Supplement.pdf]

Supplementary material 1- Survey questionnaire

This supplementary material is hosted by *Eurosurveillance* as supporting information alongside the article 'Immediate side effects of Comirnaty COVID-19 vaccine- A nationwide survey of vaccinated people in Israel, December 2020 to March 2021' on behalf of the authors who remain responsible for the accuracy and appropriateness of the content. The same standards for ethics, copyright, attributions and permissions as for the article apply. Supplements are not edited by *Eurosurveillance* and the journal is not responsible for the maintenance of any links or email addresses provided therein.

1. Did you experience one or more of the following symptoms after receiving the vaccine? Please indicate all relevant symptoms
    1. I did not experience any symptoms after receiving the vaccine
    2. Fever
    3. Fatigue
    4. Headaches
    5. Vomiting
    6. Nausea
    7. Muscle ache
    8. Joint pain
    9. Hoarseness – if yes, please contact your doctor
    10. Cough – if yes, please contact your doctor
    11. Eye irritation – if yes, please contact your doctor
    12. Facial swelling – if yes, please contact your doctor
    13. Skin rash – if yes, please contact your doctor
    14. Dizziness
    15. Significant weariness
    16. Chills
    17. Abnormal sensation in the face (numbness, tingling)
    18. Other:

2. Age: _____
3. Sex: _____

Supplementary material 2- Comparison of reporting systemic side effects after the first and second vaccine doses between pregnant women and their matched controls*

	First Dose					Second Dose				
	Pregnant women N= 1,650		Controls N=6,600			Pregnant women N=1,014		Controls N=4,052		
	N	%	N	%	P value	N	%	N	%	P value
Any Symptom	591	35.8	3,032	45.9	<.001	671	66.2	3,207	79.1	<.001
Weakness, Dizziness and Fatigue	366	22.2	1,877	28.4	<.001	542	53.5	2,631	64.9	<.001
Myalgia and Arthralgia	117	7.1	1,044	15.4	<.001	294	29	1,914	47.2	<.001
Headaches	190	11.5	1,096	16.6	<.001	343	33.8	1,867	41.6	<.001
Chills	40	2.4	316	4.8	<.001	213	21	1,477	36.5	<.001
Fever	16	1	176	2.7	<.001	146	14.4	1,170	28.9	<.001
Vomiting, Nausea and Diarrhea	101	6.1	548	8.3	0.003	166	16.4	854	21.1	<.001
Facial paresthesia	5	0.3	84	1.3	0.001	2	0.2	79	1.9	<.001
Eye irritation, Facial swelling , Rash	11	0.7	58	0.9	0.397	6	0.6	70	1.7	0.008
Hoarseness and cough	17	1	46	0.7	0.164	9	0.9	78	1.9	0.023
Other	71	4.3	356	5.4	0.074	71	7	311	7.7	0.468

*Non-pregnant controls are women respondents matched by age and ethnicity and when compared to pregnant women respondents the ratio was 4:1 respectively
